# Lef1 Promotes Foxi1-Dependent Ionocyte Specification by Airway Submucosal Gland Progenitors

**DOI:** 10.64898/2026.10.13.758987

**Published:** 2026-10-16

**Authors:** Weam Shahin, Javahir Hajibabazade, Olivia Snyder, Grace Fritz, Feng Yuan, Vitaly Ievlev, Thomas Lynch, Charles Yeaman, John F. Engelhardt

## Abstract

Pulmonary ionocytes are rare cells in submucosal gland (SMG)–containing airways thought to have specialized ion transport properties that regulate airway surface fluid and mucus composition. The origins of pulmonary ionocytes remain unclear, but their association with airway SMGs suggests they may arise from specialized progenitors in this compartment. We show that mouse glandular myoepithelial cells (GMECs) have a unique capacity to specify ionocytes enriched in the Cftr channel. The Lef1 transcription factor was both necessary and sufficient to promote GMEC specification of ionocytes in mouse trachea, 3D organoids, and/or air-liquid interface (ALI) cultures. Overexpression of Lef1 (Lef1 ^OE^ ) in GMECs produced ionocyte-enriched epithelia with enhanced Cftr-mediated chloride, bicarbonate, and fluid transport properties. Moreover, *Foxi1* knockout in Lef1 ^OE^ GMECs prevented ionocyte formation while promoting specification of Cftr-expressing secretory cells. These findings provide a progenitor relationship for the anatomical association of ionocytes with SMGs and a transcriptional framework for coordinated specification of CFTR-expressing ionocytes and secretory cells.

**Highlights:**

• Glandular myoepithelial cells give rise to pulmonary ionocytes in submucosal glands and the surface airway epithelium of mice.

• Disruption of *Lef1* in SMG progenitors decreases ionocyte specification and reduces CFTR-mediated anion transport.

• Overexpression of the Lef1 transcription factor in glandular progenitors promotes ionocyte frequency, CFTR-mediated fluid transport, and chloride and bicarbonate conductance.

•Disruption of *Foxi1* in SMG progenitors abolishes Lef1-dependent ionocyte specification and reduces CFTR-mediated anion transport.

